# Analysis of uterine corporeal mesenchymal tumors occurring after menopause

**DOI:** 10.1186/s12905-019-0714-5

**Published:** 2019-01-18

**Authors:** Yumi Ishidera, Hiroshi Yoshida, Yuka Oi, Kayo Katayama, Etsuko Miyagi, Hiroyuki Hayashi, Hiroyuki Shigeta

**Affiliations:** 1 Department of Obstetrics and Gynecology, Yokohama Municipal Citizen’s Hospital, 56 Okazawa-cho, Hodogaya-ku, Yokohama, 240-8555 Japan; 2 Department of Clinical Pathology, Yokohama Municipal Citizen’s Hospital, Yokohama, Japan; 3 Department of Obstetrics and Gynecology, Yokohama City University School of Medicine, Yokohama, Japan

**Keywords:** Leiomyoma, uterine, Leiomyosarcoma,uterine, Postmenopausal period

## Abstract

**Objective:**

Because it is difficult to diagnose accurately whether uterine corporeal mesenchymal tumors are benign or malignant before surgery, an understanding of the characteristics of patients with uterine sarcomas occurring in the postmenopausal period is required.

**Methods:**

We retrospectively reviewed the cases of women who underwent surgery for uterine mesenchymal tumors at our hospital.

**Results:**

Among 487 operated cases, 447 tumors occurred in the premenopausal period and 40 occurred in the postmenopausal period. Uterine sarcomas were observed in 5 cases (1.1%) during the premenopausal period and in 11 cases (28%) during the postmenopausal period. Among the postmenopausal patients, age, age at menopause, body mass index (BMI), tumor size, incidence of abnormal vaginal bleeding, serum tumor marker levels (cancer antigen 125, carbohydrate antigen 19–9, and carcinoembryonic antigen), and serum lactate dehydrogenase values were not significantly different between patients with benign tumors and those with malignant tumors. On the other hand, the incidence to have abnormal signal on MRI was significantly higher in patients with malignant tumors than in patients with benign tumors.

**Conclusion:**

In our hospital, the incidence of malignant tumors in women with uterine corporeal mesenchymal tumors was much higher in postmenopausal patients than in premenopausal patients. Because it is generally not easy to diagnose uterine sarcomas before surgery, surgery should be positively considered when uterine sarcomas cannot be ruled out for patients in the postmenopausal period.

## Background

Uterine corporeal mesenchymal tumors are observed in women from a wide range of age groups. Among the mesenchymal tumors of the uterine corpus, the most common neoplasms are uterine leiomyomas. They appear after menarche, typically grow during the reproductive years, and then stabilize or regress after menopause. However, there are several reports of a considerable number of patients who have undergone surgery for uterine leiomyomas in the postmenopausal period [1, 2]. When a patient presents with a uterine mesenchymal tumor, physicians should be careful in their diagnosis, because not only are benign tumors included in this classification, but malignant tumors are as well. In a study in California, it was reported that the prevalence of uterine sarcoma was 0.17–0.30% among patients who underwent hysterectomy or myomectomy, and the highest prevalence was shown for women older than 60 years [3]. In general, an accurate preoperative diagnosis as to whether a uterine tumor is benign or malignant is difficult [4]. However, it is very important to diagnose uterine mesenchymal tumors as benign or malignant, especially when a patient presents with one in the postmenopausal period. There have been few reports comparing the differences in clinical findings and incidences of malignancy between premenopausal and postmenopausal patients with uterine mesenchymal tumors. Therefore, we analyzed the clinical data of patients who underwent surgery for uterine mesenchymal tumors after menopause and compared them to those of premenopausal patients at our hospital.

## Methods

We retrospectively reviewed the medical records of patients who underwent surgery for uterine mesenchymal tumors at Yokohama Municipal Citizen’s Hospital, Yokohama, Japan, from January 2007 to December 2014. Tumors were classified histopathologically according to WHO Classification of tumors of the uterine corpus [5]. The variables tested were age, body mass index (BMI), parity, menopausal status, preoperative diagnosis, pathological diagnosis, and clinical findings. Sense of distension, hypermenorrhea, dysmenorrhea, abnormal bleeding, and abdominal pain were examined as clinical findings, and multiple symptoms were allowed to be included. Patients were divided into 2 groups; pre- and postmenopausal and the variables were compared between them. For postmenopausal patients, variables including age at menopause, BMI, tumor size, levels of several serum tumor markers, presence of abnormal bleeding, and presence of abnormal signal on magnetic resonance imaging (MRI) were further evaluated. The tumor markers examined were as follows; cancer antigen 125 (CA125), carbohydrate antigen 19–9 (CA19–9), and carcinoembryonic antigen (CEA), and serum lactate dehydrogenase (LDH) was also checked. The abnormal signal on MRI was determined when characteristic imaging features were observed in tumors such as high signal intensity on T1-weighted imaging, high signal intensity on T2-weighted imaging or restriction on diffusion-weighted imaging. Patients were divided into 2 groups; benign and malignant tumor and the variables were compared between them. Because carcinosarcomas are classified as mixed epithelial and mesenchymal tumors, they were excluded from the study. Statistical analyses were performed using JMP version 12 (SAS Institute, Cary, NC, USA). Chi-squared test was used to compare categorial variables between pre- and postmenopausal patients and benign and malignant tumor in postmenopausal patients. Student t test was used to compare continuous variables. A probability level of *P* < 0.05 was considered as statistically significant. This study was approved by the Yokohama Municipal Citizen’s Hospital institutional review board (reference no. 17–04-01) and decided to be unnecessary to obtain written informed consent from the patients for the publication of this report.

## Results

Four hundred and eighty-seven patients underwent surgery for uterine mesenchymal tumors at our hospital during the study period. Among them, 447 women (92%) were premenopausal and 40 (8%) were postmenopausal (Table 1). Malignant tumors were observed in 16 cases (3.3%) among the 487 patients, as follows: 10 cases of leiomyosarcomas, 5 cases of endometrial stromal sarcomas, and 1 case of undifferentiated sarcoma. Malignant tumors were observed in 5 cases before menopause (5/447, 1.1%) and in 11 cases after menopause (11/40, 28%).

**Table 1:** Characteristics of pre- and postmenopausal patients who underwent surgery for uterine mesenchymal tumors

	premenopausal		postmenopausal		*p*-value
Number of patients	447	92%	40	8%	
Age(yr)	44	(24–55)	62.5	(50–81)	
BMI	22.7	(16.4–42.9)	23.2	(15.4–39.4)	0.223
Nulliparity	181	40.5%	6	15.0%	< 0.001*
Pathological diagnosis					
Sarcoma(total)	5	1.12%	11	28%	< 0.001*
Leiomyosarcoma	3		7		
ESS	1		4		
Undifferentiated	1		0		
Clinical findings					
Sense of distension	199	44.5%	21	52.5%	0.407
Hypermenorrhea	247	55.3%	0	0%	NA
Dysmenorrhea	59	13.2%	0	0%	NA
Abnormal bleeding	3	0.7%	7	17.5%	0.004*
Abdominal pain	10	2.2%	2	5.0%	0.221

Values are presented as median (range), or number (%). In clinical findings, multiple symptoms were allowed to be registered. *P < 0.05. *BMI* Body Mass Index, *ESS* Endometrial Stromal Sarcoma

When we compared clinical findings between pre- and postmenopausal patients, the ratios of women who reported a sense of distension and abdominal pain were not significantly different. On the other hand, the incidence of abnormal bleeding was significantly higher in postmenopausal patients than in premenopausal patients. When we looked into the reasons for surgery in postmenopausal patients, 18 out of 40 (45%) patients underwent surgery because of suspicion of malignant tumors (Table 2). Of the remaining 22 patients, 19 patients had specific symptoms while 3 had no symptoms. Those without symptoms underwent surgery because the size of the tumors continued to increase despite being postmenopausal.

**Table 2:** The reasons for surgery in postmenopausal patients

	Total(*n* = 40)		Sarcoma(*n* = 11)		Benign(*n* = 29)	
Suspicious for malignancy	18	45%	11	100%	7	24.1%
With symptoms	11	27.5%	10	90.9%	1	3.4%
Sense of distension	6	15%	5	45.5%	1	3.4%
Abnormal bleeding	3	7.5%	3	27.3%	0	0%
Abdominal pain	2	5%	2	18.2%	0	0%
Without symptoms	7	17.5%	1	9.1%	6	20.7%
Suspicious for benign	22	55%	0	0%	22	75.9%
With symptoms	19	47.5%	0	0%	19	65.5%
Sense of distension	15	37.5%			15	51.7%
Abnormal bleeding	4	10%			4	13.8%
Back pain	1	2.5%			1	3.4%
Frequent urination	1	2.5%			1	3.4%
Myoma torsion	1	2.5%			1	3.4%
Without symptoms	3	7.5%	0	0%	3	10.3%

Values are presented as number (%). In symptoms, multiple answers were allowed to be registered

In premenopausal patients, 6 tumors were suspected to be malignant (1.3%), and this suspicion proved correct for 3 (50.0%) (Table 3). In contrast, 441 tumors were suspected to be benign, and only 2 proved to be malignant (0.45%). In postmenopausal patients, 18 tumors were suspected to be malignant, and this suspicion proved correct for 11 (61.1%); 22 tumors were suspected to be benign, and none of them proved to be malignant. The pathological diagnosis of false-positive cases included 4 cases of degenerated leiomyomas, 1 case of lipoleiomyoma, 1 case of cellular leiomyoma, 1 case of leiomyoma with massive edema, and 3 cases of typical leiomyomas.

**Table 3:** Preoperative diagnosis for pre- and postmenopausal patients who underwent surgery for uterine mesenchymal tumors

	premenopausal (*n* = 447)		postmenopausal (*n* = 40)		*p*-value
Preoperative diagnosis					
Sarcoma	6	1.3%	18	45.0%	< 0.001*
Leiomyoma	441	98.7%	22	55.0%	
Positive predictive value	3/6	50.0%	11/18	61.1%	0.336
False positive	3/6	50.0%	7/18	38.9%	0.336
False negative	2/441	0.45%	0/22	0%	0.753
Correct diagnosis rate	442/447	98.9%	33/40	82.5%	0.005*

Values are presented as number (%). *P < 0.05

Next, we compared the clinical characteristics between patients with benign and malignant tumors who underwent surgery in the postmenopausal period (Table 4). The results revealed that patient age, age at menopause, body mass index, tumor size, incidence of abnormal vaginal bleeding, serum tumor markers (CA125, CA19–9, and CEA), and serum LDH values were not significantly different between groups. On the other hand, the incidence of showing the abnormal signal on MRI was significantly higher in patients with malignant tumors than in patients with benign tumors.

**Table 4:** Comparison of the characteristics of postmenopausal patients with sarcomas and leiomyomas who underwent surgery for uterine mesenchymal tumors

	Total		Sarcoma		Benign		*p*-value
Number of patients	40	100%	11	27.5%	29	72.5%	
Age(yr)	62.5	(50–81)	66	(50–80)	60	(51–81)	0.12
Age at menopause(yr)	51	(49–57)	52	(49–55)	51	(49–57)	0.523
BMI	22.6	(15.4–39.4)	22.4	(19–27.8)	22.6	(15.4–39.4)	0.654
Tumor size(cm)	10	(2.6–30)	10	(6–30)	9	(2.6–24)	0.2
Serum CA125(U/ml)	13	(5.6–234.1)	18.3	(11–234.1)	11.5	(5.6–44.2)	0.054
Serum CA19–9(U/ml)	6.4	(1.2–303)	6	(2–19.9)	6.5	(1.2–303)	0.94
Serum CEA(ng/ml)	1.45	(0.4–6.3)	1.5	(0.5–4.9)	1.4	(0.4–6.3)	0.598
Serum LDH(IU/l)	203	(125–629)	225	(156–629)	202	(125–300)	0.08
Abnormal bleeding	7	17.5%	3	27.3%	4	13.8%	0.399
Abnormal signal on MRI	22	55%	11	100%	11	37.9%	< 0.001*

*P < 0.05

Values are presented as median (range), or number (%). *BMI* Body Mass Index, *CA125* Cancer antigen 125, *CA19–9* Carbohydrate antigen 19–9, *CEA* Carcinoembryonic antigen, *LDH* Lactate dehydrogenase, *MRI* Magnetic resonance imaging

## Discussion

To our knowledge, this is the first study to compare the characteristics of uterine corporeal mesenchymal tumors, with a specific focus on uterine sarcomas, between premenopausal and postmenopausal women. The present study showed that 8% of the patients who underwent surgery for uterine mesenchymal tumors were in the postmenopausal period. We also found that the incidence of malignant tumors was very high in postmenopausal patients (28%) and very low in premenopausal patients (1.1%).

The difference in malignancy may be because our hospital is very prudent when deciding to perform operations for postmenopausal patients with uterine mesenchymal tumors who are not suspected to have malignant tumors. However, it has been reported that the median age of patients with leiomyosarcomas is 52–57 years, and that for patients with endometrial stromal sarcomas is 50–52 years. [6, 7] Therefore, it remains interesting to determine whether there are considerable differences in the incidence of malignancy among pre- and postmenopausal patients who undergo surgery for uterine corporeal mesenchymal tumors, because patients with sarcomas are often perimenopausal.

In this study, the incidence of uterine sarcomas among women who underwent surgery for presumed uterine leiomyomas was 0.45% in the premenopausal period and 0% in the postmenopausal period. The incidence of sarcomas among patients with presumed leiomyomas has been reported to be 0.2–0.7% [8, 9]. Yilmaz et al. studied 2382 patients undergoing surgery for uterine leiomyomas and found 26 patients with sarcomas (1.1%) [4]. We could not find any reports comparing the incidence of malignant tumors between premenopausal and postmenopausal women with uterine mesenchymal tumors. However, according to a study that investigated the estimated age-stratified risk of uterine sarcomas among women undergoing surgery for presumed uterine leiomyomas, the risk was 0.17% for women 25–29 years old, and it gradually increased to 1.0% for women 75–79 years old, and decreased to 0.56% in women 80–84 years old [10]. Mao et al. studied 241,114 patients who underwent hysterectomy or myomectomy with any diagnosis and reported that the sarcoma prevalence estimates were highly dependent on age, with the lowest prevalence for women younger than 50 years of age (0.08-–0.13%) and the highest prevalence for women older than 60 years of age (between 0.36 and 1.53%). They concluded that there was a greater than 10-fold higher prevalence of uterine sarcoma among women older than 60 years of age compared with that for women younger than 50 years [3]. Therefore, careful observation is needed when performing surgery on a patient with a uterine tumor if the patient is in the postmenopausal period. On the other hand, Nagai et al. analyzed 63 patients who underwent surgery that were suspected of having uterine sarcomas [11]. Uterine sarcomas were found in 15 cases (23.8%): 3 in the premenopausal period and 12 in the postmenopausal period. Sagae et al. reported that among 106 patients with histologically proven uterine sarcomas, 65% of those with leiomyosarcomas and 75% of those with endometrial stromal sarcomas were preoperatively diagnosed as having benign leiomyomas [12]. In the current report, 14 out of 24 patients (58%) who were suspected of having uterine sarcomas were proven to have them. Therefore, the ratio of false-positive diagnoses may have a wide range of 42–76%.

Clinical characteristics, tumor markers, and diagnostic imaging are commonly used for the diagnosis of uterine sarcomas; however, it is not generally easy to diagnose them, and they are often diagnosed only after surgery for tumors suspected to be benign [10]. In this report, we found that clinical symptoms, such as the presence of abnormal vaginal bleeding and tumor size, were not useful for the diagnosis of uterine sarcomas. Nagai et al. reported that abnormal vaginal bleeding tended to be more common in patients with sarcoma than in patients with benign tumors, and no significant differences were observed in maximum tumor diameters [11]. Parker et al. reported that leiomyosarcoma is often suspected preoperatively to be malignant. They concluded that the diagnosis of uterine sarcoma should be considered in a postmenopausal woman with a pelvic mass, abnormal bleeding, and pelvic pain [13].

In this study, we found that CA125 and LDH were not useful for diagnosing uterine sarcomas. Yilmaz et al. also reported that preoperative CA125 levels were not predictive of sarcomas [4]. On the other hand, Juang et al. reported that the values of preoperative serum CA125 were significantly higher in patients with uterine leiomyosarcoma than in those with uterine leiomyomas [14]. Another report showed that the serum LDH value significantly predicted uterine sarcomas, while CA125 did not [11]. From these observations, CA125 may not be useful for the diagnosis of uterine sarcomas and LDH may be useful, but this remains controversial. Duk et al. reported that uterine sarcoma cells were completely negative for CA125. They speculated that the source of the elevated serum CA125 levels in patients with uterine sarcoma may be stimulated mesothelial cells [15].

MRI has proven to be one of the most useful imaging modalities in making the preoperative differentiation between tumors, but even with MRI, it is difficult to distinguish between uterine sarcomas and uterine leiomyomas with degeneration [16–18]. In this study, all the postmenopausal patients of uterine sarcoma had abnormal signal on MRI. In contrast, abnormal signal was shown in 11 out of 29 patients (37.9%) of leiomyoma. Although the incidence of abnormal signal on MRI was significantly higher in patients with uterine sarcomas than in uterine leiomyomas, careful consideration is needed in diagnosing sarcoma. Increased uptake on positron emission tomography (PET) with [^18^F]fluorodeoxyglucose (FDG) has been shown in leiomyosarcomas [19]; however, it has also been reported that FDG PET alone cannot be used to differentiate leiomyosarcomas from leiomyomas [20]. Although it has been reported that estrogen receptor (ER) expression coupled with glucose metabolism using ER imaging agents for PET, such as 16α-[^18^F]-fluoro-17β-estradiol and FDG PET, is useful for the differential diagnosis of leiomyomas and leiomyosarcomas, there were still some overlapping cases [21]. Kawamura et al. tried to diagnose uterine sarcomas by transcervical needle biopsies and a scoring system that included the mitotic index, cytologic atypia, and coagulative tumor cell necrosis [22]. They suggested that the scoring system was effective; however, it was not definite. They recommend surgery when uterine sarcoma cannot be excluded.

## Conclusions

When postmenopausal patients undergo surgery for uterine corporeal mesenchymal tumors, their incidence of malignancy is much higher than that for premenopausal patients. Because it is not generally easy to diagnose uterine sarcoma before surgery, physicians should consider surgery positively when uterine sarcomas cannot be ruled out, especially for postmenopausal patients.

## Abbreviations

BMI: body mass index
CA125: cancer antigen 125
CA19–-9: carbohydrate antigen 19–-9
CEA: carcinoembryonic antigen
ER: estrogen receptor
FDG: fluorodeoxyglucose
LDH: serum lactate dehydrogenase
MRI: magnetic resonance imaging
PET: positron emission tomography
